# Laceration of the Perineum

**Published:** 1907-09-21

**Authors:** 


### Gynæcology.

### LACERATION OF THE PERINEUM.

It is impossible to estimate with any certainty the percentage of labours in which perineal laceration occurs, but it must be conceded by all that the number of primiparous patients who are delivered without some tearing is very small indeed. The tendency to laceration depends very much on the age of the individual, as well as on other factors, such as the size of the child and the character of the uterine contractions. Primiparæ over thirty practically never escape without a laceration if labour occurs at full term. From the clinical standpoint two causes of perineal laceration stand out pre-eminently. First, the extension movement by which the head is born; second, the precipitate character of the labour which sometimes occurs when the uterine contractions are very powerful and the child under the average size. The first is by far the commoner cause of severe tears, although the latter is more liable to produce very extensive injuries when it does occur. It is clear that when a small child is rapidly driven down by powerful contractions, there is no time for gradual dilatation of the vulval outlet. If, then, the contractions are sufficiently strong, the fœtal head is driven through the perineal tissues, and very severe lacerations may result.

The common cause, however, is the extension movement of the head, whereby a much greater circumference of the head is substituted for a smaller one, with consequent injury to the maternal soft parts if they are already stretched to their utmost limit. The suboccipito-bregmatic circumference measures from 11 to 12 inches, whilst the occipito-frontal is usually 14 inches, the latter being substituted for the former at the moment of birth by the extension movement. Very much the same result is produced by unreduced occipito-posterior presentation, as it is the occipito-frontal circumference which then distends the perineum. It is often stated that the shoulders are responsible for severe perineal tears, but this is seldom the case. What the shoulders sometimes can accomplish is to make much worse a laceration which has been already commenced by the head; but even that is not a very common occurrence. It becomes of importance to consider how these injuries may be prevented, or at least limited. The ideal method for the head to be born would be flexed to the utmost, so that the suboccipito-bregmatic circumference might pass the outlet, but this is impossible, because the head has to pass around the symphysis pubis, the presenting part following strictly the line of the curve of Carus. The amount of extension, however, and the time at which it occurs may be controlled to some extent, and by such control the amount of perineal laceration may be limited. The best way to do this is by means of pressure of the thumb and second finger upon the forehead of the child, placed on either side of the anus of the mother. Firm compression in this way does not at all impede the stretching of the perineal tissues, but helps to keep the head flexed as long as possible, and allows extension to occur gradually. By the same manœuvre the head may be held at the outlet between the uterine contractions, and may be slowly squeezed out from behind forwards by the fingers alone. This procedure permits of a slower delivery of the head in a more favourable position than would be possible during a powerful contraction. The old method of "preserving" the perineum by compression with the palm of the hand was wrong in principle, because it helped to prevent stretching of the perineal tissues. The statement that the application of the forceps will sometimes (often, according to some) save the perineum, is absolutely untrue in the wide sense, because a forceps extraction is rapid, the extension movement cannot be controlled, and the mere added thickness of the blades increases the circumference which has to pass the outlet. In a limited sense, however, the forceps can do something to prevent the complicated lacerations and great bruising which are apt to occur when a head is long delayed at the outlet. Everyone is familiar with the case in which the contractions are weak and the pelvic floor unyielding. Long delay of the head on the perineum in such a case means bruising of the posterior vaginal wall with subsequent boring and tearing of the perineal body, without any external skin laceration. This preliminary "boring-out" of the perineal body can certainly be prevented by a timely forceps extraction, but that some ordinary antero-posterior laceration would be prevented with the forceps in such a case is quite untrue as a rule.

It might be supposed that it is superfluous to insist upon the immediate repair of all perineal lacerations, however small, but it is a fact that even in these days many such injuries go unsutured. It is quite common to hear the statement "that torn perinea never occurs in the practice of some individuals." We can only say that these individuals would see plenty if they looked for them. Again, it is a common belief that a torn perineum will unite if the legs are tied together; this is an absolute fallacy. It will granulate and heal in time, but the surfaces will never unite by first intention. Therefore we must insist that these injuries ought to be sutured at once, if the future comfort of the patient is to be considered. It is by no means easy to suture a perineum well, without an anæsthetic, and with the parts bathed in blood. The difficulties, however, can be minimised by performing the operation with the patient in the dorsal position and the legs held up; a simple way to do this is to tie one leg to the end of the bed and get the nurse to hold the other if no crutch is available. Silk-worm gut is the best all-round material for the deep sutures, whilst any vaginal tear may be brought together with a continuous catgut suture. The catheter is unnecessary and meddlesome afterwards if the patient can pass urine herself, and it is better to get the bowels open early, at the end of twenty-four hours by castor oil, than to leave them three or four days with the possibility of the passage of a very hard motion.

